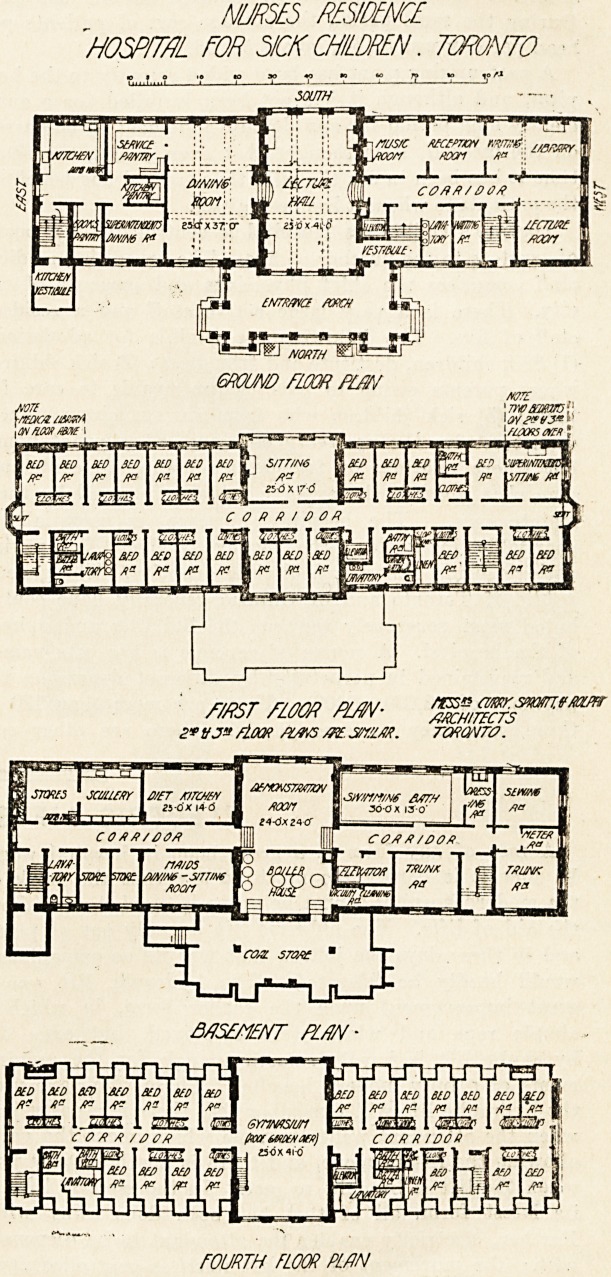# The Hospital for Sick Children, Toronto

**Published:** 1910-05-07

**Authors:** 


### SPECIAL ARTICLES.

### THE HOSPITAL FOR SICK CHILDREN, TORONTO.

Whereas the General Hospital in Toronto, described in a former article, is an old institution and inadequate to the needs of the community in all respects, the Toronto Children's Hospital is up to date in every particular, and is esteemed a model of its kind by all who have seen it. A hospital for sick children was first established in Toronto on quite a humble scale by two philanthropic ladies in 1875. For some time it continued in a small way, the parents of infants and young children being somewhat prejudiced against hospital treatment for their offspring. This sentiment, due to ignorance, wore off, and after the lapse of a few years it was found that the accommodation provided was not nearly equal to the calls made upon it by the population of the city and surrounding district. Up to the year 1891 the work of the hospital was carried on in what may be termed more or less makeshift buildings, but in that year a new and complete building was opened. The trustees of the hospital were in possession of an excellent site on College Street, and there the new hospital was erected. The city gave a handsome grant of money towards the expenses, and voluntary contributions flowed in generously, so that those responsible for the planning and erection of the building were enabled to carry out the most modern and ambitious designs in children's hospital construction. The result has been most satisfactory.

Victoria Hospital for Sick Children is situated on the south side of College Street, adjoining the large plot of land on which the proposed new general hospital for Toronto is about to be built, and almost opposite the University. It has a frontage of 150 feet on College Street, and extends back 105 feet. The plan of the building is in the form of an E, with the straight side on the north, the arms running south and enclosing an area protected from the winds on the west, north, and east sides, and open to the south; verandahs are placed on all sides of this area, so that beds may be carried out upon them from the different wards. The large wards are in the east and west wings, and the smaller wards and administration departments in the front portion of the building. The building has six floors. The lowest or sub-basement contains boiler room, cold storage, fresh-air passages, and heating chambers. The basement or lower floor, which is only 2 feet below College Street, contains kitchen, pantries, dining, linen, and sewing rooms, dispensary department, receiving room, and laundry. The ground floor contains board room, the matron's apartments, visitors' rooms, two wards 21 feet square, two wards 21 feet by 54 feet; pantries, bath and dressers' room, etc. The first floor contains two wards 21 feet square, two wards 21 feet by 54 feet, one ward 41 feet by 21 feet, several small wards, pantries, bath and dressers' rooms, etc. The second floor contains one ward 21 feet by 54 feet, convalescent ward 41 feet by 21 feet, and offices. A portion of this floor is isolated from the remainder of the building, and is used for infectious cases. It contains five wards and offices, and is reached by means of a fireproof staircase. The top floor contains bedrooms for the resident staff, *x*-ray department, school room and offices. There are twenty private wards, and an excellent operating theatre, with ample accommodation for students. There is also a fully equipped bacteriological department.

All wards throughout the building, and many of the other rooms, are heated on the indirect principle. The heating appliances have been so arranged that the building can be heated by gravity, exhaust, or high-pressure steam, and the building is lighted by electricity throughout. The hospital can take in 195 patients, but the usual average is between 140 and 150. It is a roomy, spacious building, and a feature that is especially noticeable is the roofed verandah opening from each large ward, into which the little ones can be taken when the weather is fine—these really almost double the capacity of the hospital. Another point worthy of mention is that the dressing of cases is never done in the wards themselves, the patients are wheeled into a room constructed for the purpose opening from the ward. The majority of the cots can be raised or lowered at the will or need of the medical man or nurse.

The schoolroom is another feature of much interest. Of course, it is intended for convalescents, and the work consists of classes in kindergarten and elementary education, under the supervision of a competent teacher put in by the Public School Board of Toronto. The school was commenced in 1892, and during 1908 there were 221 pupils.

During the hospital year of 1908, 1,245 children were admitted as in-patients, of whom 698 were boys and 547 were girls. In answer to urgent calls the hospital opened an infant ward in 1906. The total attendance at the outdoor department in 1908 was 10,663. Of these patients treated there were 466 eye, ear, nose, and throat cases, 789 orthopædic, and 9,408 general cases. In 1908, 368 patients were admitted from 241 placed in Ontario outside Toronto. During the same period the average cost of patients per head per day was $1.37\frac{1}{4}$, about 5s. 9d.

A pasteurising plant has been added recently to the hospital, and although it has not been installed for a sufficiently long period for any definite judgment to be passed on the method, it is thought that even during the short time it has been in use the milk thus treated has benefited the patients to whom it has been given.

Toronto's Children's Hospital is controlled by a board of trustees to the number of five. The consulting medical staff comprises the chief physicians and surgeons of the city. There is a very large active staff and a resident staff of five. The following are eligible for admission: (1) Sick children, destitute and friendless; (2) sick children whose parents owing to poverty are unable to care for them; (3) sick children who from various circumstances cannot receive the necessary care and attention at home, but whose relations or friends are willing to contribute somewhat towards the expense actually incurred by their maintenance. The hospital, however, is essentially a charitable institution, the money received for paying patients being almost a negligible quantity. The general public of Toronto and of Ontario generally have contributed most generously towards the building and upkeep of the hospital. A source of revenue is the cots named and maintained in perpetuity by different donors on the payment of $2,000 (£400). There are more than 120 of these, and they increase yearly. There are other cots which are named and maintained for a year by the payment of $100 (£20). Of these there are nearly seventy. One citizen of Toronto, Mr. J. Ross Robertson, proprietor of the *Evening Telegram*, has been the main agent in establishing the Children's Hospital and its mainstay since its establishment. He has given largely towards the erection and maintenance of the building, has built solely at his own expense a convalescent home, and has built and furnished a nurses' home. Mr. Robertson has, moreover, given much of his time and the benefit of his acute business qualities to the furtherance of schemes calculated to assist the hospital.

In connection with the Children's Hospital there is a convalescent home, as there should be in connection, not only with children's hospitals, but with all hospitals.

In 1883 the city of Toronto granted a plot of land upon an island in Lake Ontario, near the city, for the erection of a convalescent home. Mr. Robertson erected the first building, he added a wing in 1886; and in 1891 caused the entire building to be remodelled and the accommodation more than doubled at the cost of over $30,000 (£5,000). The Convalescent Home occupies a site on the west point of Toronto Island, about two miles distant from the mainland. It is an exceedingly picturesque building, constructed mainly of wood, is spacious, airy, well ventilated, and well lighted; indeed, in every particular admirably adapted to the purpose for which it was built. It has balconies for every ward and beds for 150 patients. At the end of May of each year 100 or so patients from the mother hospital in College Street are removed to the Lakeside Home, the name by which the convalescent home is known. During the months of June, July, August, and September in 1908, 350 patients had each some weeks at this home. The broad, open balconies served as wards, and 100 patients slept on cots on these balconies for the four months that this home on the lake was open. Cots are endowed, named, and maintained in perpetuity in the Lakeside Home by the payment of $500 (£100). Of such cots there are four. Cots are also maintained in this home by an annual payment of $25 (£5).

## Figures and Tables

**Figure f1:**